# Unraveling the Clinical Complexity of Thyrotoxic Periodic Paralysis: A Case Report

**DOI:** 10.7759/cureus.66195

**Published:** 2024-08-05

**Authors:** Gowri Renganathan, Sudhagar Thangarasu, Baljinder Singh, Simrandeep K Brar

**Affiliations:** 1 Internal Medicine, Texas Tech University Health Sciences Center El Paso Paul L. Foster School of Medicine, El Paso, USA; 2 Neurology, New York University (NYU) Langone Health, New York, USA

**Keywords:** graves disease, channelopathy, periodic paralysis, endocrine emergency, muscle weakness, hyperthyroidism, hypokalemia, thyrotoxic periodic paralysis

## Abstract

Thyrotoxic periodic paralysis (TPP) is a clinical condition characterized by hypokalemia, muscle paralysis, and hyperthyroidism. TPP can be challenging to diagnose due to its low disease prevalence and the similarity of paralysis to other common conditions. Through this case report, we highlight the importance of considering hyperthyroidism as a cause of recurrent attacks of muscle paralysis, particularly in the setting of other signs of hyperthyroidism. A 32-year-old Hispanic man with a history of recurrent episodes of muscle weakness presented to the hospital with the acute onset of bilateral lower extremity weakness and an inability to ambulate. Additionally, the patient was experiencing symptoms of hyperthyroidism, including heat intolerance, weight loss, anxiety, and tremors. Lab evaluation showed hypokalemia, and the thyroid panel indicated hyperthyroidism due to Graves disease. His symptoms resolved after the replacement of potassium orally and intravenously, and he was discharged home on methimazole and propranolol. The presented case emphasizes that endocrinological and metabolic causes should be considered in the differential diagnosis of acute flaccid paralysis. The symptoms of hyperthyroidism can be subtle in many cases, which can make the diagnosis very challenging.

## Introduction

Thyrotoxic periodic paralysis (TPP) is a rare medical condition characterized by a triad of hypokalemia, muscle paralysis, and hyperthyroidism. It is a potentially life-threatening complication of hyperthyroidism that can involve the respiratory system and requires prompt recognition and treatment. While its prevalence is low among Caucasians, it is approximately 10 times more common in the Asian population, suggesting a genetic predisposition [1,2]. TPP often goes unnoticed due to its low disease prevalence, the subtlety of clinical hyperthyroidism symptoms, and the similarity of paralysis to other common conditions. Through this case report, we elucidate the pathophysiology, clinical presentation, diagnostic approach, and management strategies for TPP, highlighting the significance of prompt intervention to prevent complications and ensure optimal patient outcomes. This article further highlights the importance of suspicion of hyperthyroidism as a cause for the recurrent attacks of proximal muscle weakness in the setting of systemic symptoms such as tachycardia, weight loss, and proptosis. It is critical to differentiate this condition from hypokalemic periodic paralysis, which can clinically mimic the presentation.

## Case presentation

A 32-year-old Hispanic man with a history of recurrent episodes of proximal and distal muscle weakness presented to the hospital with the acute onset of bilateral lower extremity weakness and an inability to walk. He woke up with this sudden onset of lower extremity weakness, which prompted his visit to the emergency room. He had experienced five similar episodes over the past six years that had resolved completely after intravenous and oral potassium supplementation. His only home medication included 40 milliequivalents (mEq) of potassium chloride (KCl) daily, which he had not taken for the past three days as he had run out of the medication. He reported experiencing heat intolerance, 10 lb weight loss despite having a good appetite, and episodes of anxiety and tremors for the past two months. No other family members had similar symptoms. He had no history of using alcohol, illicit drugs, or diuretics.

On a comprehensive physical examination, tachycardia and mild bilateral proptosis were noted. Mental status was normal. His speech was clear, and his comprehension was intact. On the Medical Research Council (MRC) scale, his muscle strength was 3/5 bilaterally for hip flexion, knee flexion was 3/5, knee extension was 4/5, ankle dorsiflexion was 4/5, and plantar flexion was 5/5. The cranial nerves were intact. The upper extremities showed a strength of 5/5. Coordination was normal. Fine, intentional tremors were noticed during the examination. The serum potassium level was found to be 2.7 mEq/L during the acute attack (normal range: 3.5-5.1 mEq/L). An electrocardiogram (ECG) showed sinus tachycardia. The patient was given 40 mEq of intravenous KCl, followed by another 40 mEq of KCl orally. In the next three hours, his lower extremity strength recovered to 5/5 bilaterally, and he regained the ability to walk. Repeat labs showed a rapid response to KCl with a potassium level of 5.8. Due to his symptomatology consistent with hyperthyroidism, a complete thyroid panel was ordered, and the results indicated hyperthyroidism due to Graves disease. The TSH (thyroid-stimulating hormone) level was 0.008 IU/mL, thyroid-stimulating hormone receptor antibody (TRAb) was 49% (reference range: ≤16%), total thyroxine (T4) was 16.4 mcg/dL (reference range: 4.8-10.4), and total triiodothyronine (T3) was 279 ng/dL (reference range: 76-181). The urine potassium creatinine ratio was <1. The thyroid-stimulating immune globulin was 139% (reference range: <140% baseline).

Additionally, an ultrasound of the thyroid gland demonstrated a diffusely enlarged thyroid gland with increased vascularity. Notably, the patient had borderline normal corrected calcium levels and slightly elevated phosphate levels, which were of unknown significance (Table 1). He was started on propranolol 20 mg per oral (PO) twice daily and methimazole 30 mg PO daily. Serial measurements of potassium remained within normal limits without potassium replacement. The patient was discharged home with prescriptions for methimazole and propranolol, along with instructions to follow up with an endocrinologist outpatient.

**Table 1:** Labs and imaging TSH: thyroid-stimulating hormone; T3: triiodothyronine; T4: thyroxine

Lab Test	Value	Reference Range
Serum potassium	2.7 mEq/L	3.5-5.1 mEq/L
Calcium	8.5 mEq/L	8.5-10.1 mEq/L
Phosphate	5.3 mg/dL	2.5-4.9 mg/dL
TSH	0.008 uIU/mL	0.55-4.78 uIU/mL
Total T3	279 ng/dL	76-181 ng/dL
Total T4	16.4 mcg/dL	4.8-10.4 mcg/dL
Thyroid-stimulating hormone receptor antibody	49%	<16%
Thyroid-stimulating immune globulin	139%	<140% baseline
Ultrasound thyroid gland	Enlarged thyroid gland. Increased vascularity to the thyroid gland	-

## Discussion

This section delves into the pathophysiological mechanisms underpinning TPP, emphasizing the dysregulation of potassium homeostasis mediated by excess thyroid hormones. It elucidates the interplay between hormonal, genetic, and environmental factors contributing to the development of TPP, as well as the differential diagnosis and diagnostic criteria essential for accurate identification. TPP is commonly reported in Asians and is rare in Hispanics and Whites. It is characterized by the triad of recurrent transient muscle paralysis that typically occurs at night, acute hypokalemia without a total body potassium deficit, and thyrotoxicosis [3]. The condition affects males to a much greater extent than females, with a male-to-female ratio ranging from 17:1 to 70:1, and it is attributed to the role of male hormones on sodium-potassium ATPase (Na+/K+-ATPase) activity [3,4]. The majority of patients, approximately 80%, experience the onset of symptoms between the ages of 20 and 39 [3,5]. The onset of TPP is often sudden and may be triggered by factors such as a high-carbohydrate meal, strenuous exercise, emotional stress, or even rest after exertion [6,7]. Additional contributing factors include trauma, cold exposure, infection, alcohol abuse, menstruation, and medications such as steroids, fluoroquinolones, or diuretics [1,6]. Muscle weakness primarily affects the lower extremity proximal muscles, but it can progress rapidly to involve the distal muscles and even the respiratory and bulbar muscles, posing a risk of respiratory failure [2,8,9]. The paralysis usually lasts up to 96 hours and frequently resolves in the reverse order from which it occurred [1].

The pathophysiology of TPP involves alterations in potassium handling in skeletal muscles. It is believed that excess thyroid hormones, such as T4, increase the activity of the Na+/K+-ATPase pump in the sarcolemma, leading to an intracellular shift of potassium ions. This shift results in a state of hypokalemia, which disrupts the normal electrical potentials of muscle cells and leads to muscle weakness and paralysis [4]. The HLA-DRW8 association raises the possibility that the basic defect may be genetically determined; however, the Na+/K+-ATPase pump plays a major role in the pathophysiology of TPP [10]. Insulin, β-adrenergic catecholamines, and thyroid hormone stimulate the Na+/K+-ATPase pump, which causes a shift in potassium into the cells and results in hypokalemia without affecting the potassium level in the body as a whole. This explains the resolution of the symptoms with the shift of potassium to the extracellular space [11,12]. Insulin acts synergistically with thyroid hormone to drive potassium into the cell; hence, attacks are often precipitated by a heavy meal, and hyperinsulinemia is a common laboratory finding during the acute episode [6,7]. Advances in understanding the pathophysiology of TPP have identified mutations in the inwardly rectifying potassium channel Kir 2.6. Loss-of-function mutations in the genes encoding these channels (Kir2.6) can prevent the flow of potassium out of skeletal muscle cells, disrupting potassium homeostasis [7,13,14]. However, the exact genetic pathophysiology of TPP is undetermined; further genetic testing in patients with complex clinical presentations can be helpful to rule out channelopathies such as hypokalemic periodic paralysis.

Diagnosis is often confirmed by low serum potassium levels and thyroid function testing, indicating a hyperthyroid state. Acute thyrotoxicosis and hypokalemia alone, however, may not indicate TPP, as individuals with hyperthyroidism may also have chronic hypokalemia brought on by laxative usage, mineralocorticoid excess status, diuretics, or concurrent renal tubular diseases. TPP can be diagnosed in such scenarios by evaluating renal K+ excretion, acid-base state at presentation, and the quantity of KCl needed to treat hypokalemia [7]. There have been reports of patients with increased T3 levels and normal T4 levels, particularly in cases of TPP due to Graves disease (T3 thyrotoxicosis) [15]. Familial hypokalemic periodic paralysis is a common differential and can be ruled out by laboratory findings of mild hypophosphatemia and hypomagnesemia [16,17]. Familial periodic paralysis typically manifests at an earlier age than TPP. Hence, a later age of onset can suggest TPP, although some cases of adolescents with TPP have been reported [5]. If the findings are inconclusive, a urine calcium-to-phosphate ratio >1.7 can be used to distinguish TPP from familial hypokalemic periodic paralysis [18].

Treatment involves potassium replacement, preferably administered intravenously for faster potassium restoration and resolution of paralysis [19]. It is important to note that hypokalemia and muscle paralysis result from intracellular shifting of potassium rather than a total body potassium deficit. Therefore, excessively aggressive correction of potassium levels can lead to rebound hyperkalemia. Rebound hyperkalemia usually occurs if the patients are treated with >90mEq of KCl within the first 24 hours [1,17]. Non-selective beta-blockers can also be used, as they rapidly reverse the initial intracellular shift of potassium by blocking Na+/K+-ATPase activity [1]. The effective treatment options during an acute attack are low-dose potassium supplementation and beta-blocker therapy. The long-term goal is to prevent further attacks, which require definitive treatment for hyperthyroidism, such as radioactive ablation, anti-thyroid medications, or thyroidectomy, as in this patient suffering from Graves disease.

The transient nature of the events and the rarity of the disease in Western populations can contribute to delayed diagnosis. The signs and symptoms of hyperthyroidism may be subtle in these patients, making the diagnosis challenging. Thyroid function testing should be included as part of the diagnostic workup in patients presenting with muscle weakness, particularly middle-aged Asian men, due to its higher prevalence in this demographic [20].

The presented case highlights the importance of considering endocrinological and metabolic causes in the differential diagnosis of acute flaccid paralysis. Unlike other forms of muscle paralysis, TPP can be relatively easy to treat with rapid potassium replacement, leading to complete recovery of muscle strength and the potential prevention of dangerous complications associated with hypokalemia, such as cardiac arrhythmias and respiratory muscle paralysis. Additionally, it is crucial to recognize and avoid common triggers that precipitate paralytic attacks. Treatment of hyperthyroidism is curative for TPP.

## Conclusions

The manuscript concludes by reaffirming the clinical significance of TPP as an endocrine emergency necessitating prompt recognition and intervention. It underscores the importance of heightened clinical suspicion, comprehensive diagnostic evaluation, and targeted management strategies in optimizing patient outcomes and mitigating the risk of recurrence. Additionally, it also emphasizes the need for further research to elucidate the genetic and environmental determinants of TPP, thereby facilitating more effective prevention and management strategies.
